# Long-Term Outcomes of Mechanical Mitral Valve Replacement: A Comparison of Four Valve Types

**DOI:** 10.3390/jcm15041633

**Published:** 2026-02-21

**Authors:** Amr A. Arafat, Fatimah A. Alhijab, Monirah A. Albabtain, Musab Kiddo, Rwan Alghamdi, Saud Alshehri, Ismail M. Alnaggar, Mostafa A. Shalaby, Huda H. Ismail, Khaled A. Alotaibi

**Affiliations:** 1 Adult Cardiac Surgery Department, Prince Sultan Cardiac Center, Riyadh 12233, Saudi Arabia; falhijab@pscc.med.sa (F.A.A.); mkiddo@pscc.med.sa (M.K.); ralghamdi@pscc.med.sa (R.A.); saalshehri@pscc.med.sa (S.A.); ielnaggar@pscc.med.sa (I.M.A.); mshalaby@pscc.med.sa (M.A.S.); hismail@pscc.med.sa (H.H.I.); kaalotaibi@pscc.med.sa (K.A.A.); 2 Research and Innovation Institute, Ministry of Defense Health Services, Riyadh 12426, Saudi Arabia; 3 Research Department, Prince Sultan Cardiac Center, Riyadh 12233, Saudi Arabia; malbabtain@pscc.med.sa; 4 Cardiothoracic Surgery Department, Cairo University, Cairo 11562, Egypt

**Keywords:** mitral valve replacement, heart valve prosthesis, mechanical heart valves, stroke, survival, ventricular remodeling, bileaflet mechanical valves

## Abstract

**Background:** The choice of mechanical prosthesis for mitral valve replacement (MVR) is critical, yet data comparing long-term outcomes across different valve types are still needed. This study aimed to compare the long-term clinical and echocardiographic outcomes of four distinct mechanical mitral valve prostheses. **Methods:** We retrospectively analyzed 431 patients who underwent mechanical MVR between 2009 and 2022 with one of four valve types: Carbomedics (n = 112), Bicarbon (n = 176), ATS (n = 89), or On-X (n = 54). A competing risk regression model was used to identify predictors of a composite endpoint (valve thrombosis, reoperation, stroke, pulmonary embolism, and major bleeding), accounting for all-cause mortality. Longitudinal echocardiographic data were analyzed using linear mixed-effects models. **Results:** The median follow-up was 62 months. The cumulative incidence of the composite endpoint at 10 years was 14% for the On-X valve, 12% for the Bicarbon valve, 9.5% for the Carbomedics valve, and 7% for the ATS valve. After adjusting for confounders, the type of valve prosthesis was not significantly associated with the composite endpoint. Significant predictors of adverse events included coronary artery disease (Sub-distribution Hazard Ratio [SHR] 2.70, *p* = 0.023), peripheral artery disease (SHR 6.29, *p* = 0.007), and smaller valve size (SHR 0.87, *p* = 0.037). No significant difference in overall survival was observed between the groups (log-rank *p* = 0.904). All valve types were associated with favorable LV remodeling. The Carbomedics group showed the greatest reduction in left ventricular end-diastolic diameter, likely reflecting regression to the mean given the larger baseline ventricular dimensions in this group. **Conclusions:** The type of mechanical mitral valve did not significantly influence long-term thromboembolic and bleeding events or overall survival. Patient-specific factors and valve size were the primary determinants of adverse outcomes. The observed differences in ventricular remodeling may warrant further investigation.

## 1. Introduction

Mitral valve disease represents a significant and growing global health burden, especially in developing countries where rheumatic heart disease is still prevalent in the young population [1,2]. While medical management can alleviate symptoms in the early stages, surgical intervention remains the definitive treatment for severe, symptomatic mitral valve disease [3]. Mitral valve repair is generally preferred when technically feasible; however, in cases of extensive valve damage, severe calcification, or certain etiologies such as rheumatic heart disease, mitral valve replacement (MVR) becomes the preferred surgical option [4,5]. The choice of prosthesis for MVR is a critical decision that involves a trade-off between durability and the need for lifelong anticoagulation [6].

Bioprosthetic valves have the advantage of not requiring long-term anticoagulation; however, they are prone to structural valve deterioration over time, often necessitating reoperation [6,7]. In contrast, mechanical valves offer excellent durability and are designed to last a lifetime. This durability, however, comes at the cost of a higher risk of thromboembolic events. The management of anticoagulation itself carries a risk of bleeding complications, and patients require regular monitoring to maintain their international normalized ratio (INR) within a narrow therapeutic range [8]. The evolution of mechanical heart valves has been a remarkable journey of biomedical engineering, from the early caged-ball and tilting-disk designs of the 1960s to the modern bileaflet valves that are now the standard of care [9]. The introduction of bileaflet valves in the late 1970s represented a significant advancement, offering improved hemodynamics, lower thrombogenicity, and quieter operation. Since then, several other bileaflet mechanical valves have been developed, each with subtle design variations in leaflet shape, pivot mechanisms, and housing materials, all aimed at optimizing blood flow and reducing the risk of complications [9].

Despite the widespread use of modern bileaflet mechanical valves for several decades, there is still a need for long-term comparative data on the clinical and echocardiographic outcomes of different valve types. While all contemporary bileaflet valves have demonstrated excellent overall performance, it is plausible that subtle design differences could translate into variations in long-term outcomes, such as the incidence of thromboembolic events, bleeding complications, or the degree of left ventricular remodeling. The four valve types examined in this study, Carbomedics (Corcym S.R.L., Saluggia, Italy), Bicarbon (LivaNova PLC, Saluggia, Italy), ATS (Medtronic, Minneapolis, MN, USA), and On-X (On-X Life Technologies, Inc., Artivion, Inc., Austin, GA, USA), are all established bileaflet prostheses that have been used extensively in clinical practice. However, head-to-head comparisons of their long-term performance in the mitral position are limited. Therefore, the aim of this study was to compare the long-term clinical and echocardiographic outcomes of these four commonly used mechanical mitral valve prostheses in a large, single-center cohort.

## 2. Materials and Methods

This study was conducted and reported in accordance with the Strengthening the Reporting of Observational Studies in Epidemiology (STROBE) guidelines for cohort studies [10].

### 2.1. Study Design and Population

We conducted a retrospective, single-center, observational cohort study involving patients who underwent primary or redo mechanical MVR between 2009 and 2022 at Prince Sultan Cardiac Center, Riyadh, Saudi Arabia. A total of 431 consecutive patients were included and stratified into four groups based on the type of bileaflet mechanical prosthesis implanted: Carbomedics (n = 112), Bicarbon (n = 176), ATS (n = 89), and On-X (n = 54). The study was approved by the Institutional Review Board, and the requirement for individual patient consent was waived due to its retrospective nature (Approval #1698, dated 3 June 2024).

### 2.2. Variables and Data Sources

#### 2.2.1. Data Collection

Patient data were retrospectively collected from the institutional electronic health records and surgical database. This included preoperative, intraoperative, and postoperative data. Follow-up information was obtained from outpatient clinic records.

#### 2.2.2. Baseline and Operative Variables

Preoperative patient characteristics, including demographics, comorbidities, and clinical status, were collected. Operative risk was calculated for each patient using the European System for Cardiac Operative Risk Evaluation (EuroSCORE) II, and its component variables were defined according to the official EuroSCORE II model definitions [11]. Intraoperative variables included cardiopulmonary bypass (CPB) time, aortic cross-clamp time, and the size of the implanted prosthesis.

### 2.3. Operative Techniques

All procedures were performed through a median sternotomy with CPB and moderate hypothermia. After aortic cross-clamping and antegrade cardioplegia, the mitral valve was exposed through a trans-atrial approach in most cases. The choice of valve prosthesis was at the discretion of the operating surgeon, subject to institutional contracts and valve availability. In general, the subvalvular apparatus was preserved whenever feasible, particularly the posterior leaflet, to maintain left ventricular geometry and function. The extent of leaflet resection and annular decalcification was determined by the underlying pathology.

### 2.4. Outcomes

The primary outcome was a composite endpoint of major adverse events, including valve thrombosis, reoperation, stroke, pulmonary embolism, and major bleeding. Bleeding events were defined and classified according to the Bleeding Academic Research Consortium (BARC) criteria, with BARC types 3–5 considered major bleeding [12]. Valve thrombosis was an echocardiographic diagnosis. Stroke was defined as a new focal neurological deficit lasting more than 24 h, confirmed by neuroimaging.

Secondary outcomes included all-cause mortality, heart failure readmission, recurrence of New York Heart Association (NYHA) class III/IV, and longitudinal echocardiographic parameters. Echocardiographic follow-up included measurements of left ventricular ejection fraction (LVEF), left ventricular end-diastolic dimension (LVEDD), and left ventricular end-systolic dimension (LVESD).

### 2.5. Anticoagulation Quality

The quality of anticoagulation therapy with vitamin K antagonists was assessed by calculating the time in therapeutic range (TTR) for each patient. The TTR was calculated using the Rosendaal linear interpolation method, which assumes a linear relationship between two consecutive International Normalized Ratio (INR) measurements [13]. The target therapeutic range for all patients was an INR of 2.5 to 3.5.

### 2.6. Statistical Analysis

All statistical analyses were performed using Stata BE version 18.0 (StataCorp LLC, College Station, TX, USA). A *p*-value of less than 0.05 was considered statistically significant for all analyses.

#### 2.6.1. Data Presentation and Baseline Comparisons

Continuous variables were assessed for normality using the Shapiro–Wilk test. Normally distributed data are presented as mean and standard deviation (SD), while non-normally distributed data are presented as median and interquartile range (IQR). Categorical variables are presented as counts and percentages. For comparisons of baseline characteristics and outcomes among the four valve groups, the one-way analysis of variance (ANOVA) was used for normally distributed continuous variables, and the Kruskal–Wallis test was used for non-normally distributed continuous variables. The Chi-square test was used for categorical variables. In cases where the expected cell count was less than five, Fisher’s exact test was used instead.

#### 2.6.2. Analysis of Long-Term Outcomes

To identify predictors of the composite endpoint, a competing-risks regression analysis using the Fine and Gray model was performed. This method was chosen to appropriately handle the competing risk of all-cause mortality. All baseline and operative variables, in addition to TTR, were entered into a forward stepwise competing-risk regression. The final model included variables with a significant *p*-value (<0.05), in addition to the valve types. The results from this analysis are presented as sub-distribution hazard ratios (SHRs) with corresponding 95% confidence intervals (CIs). Time-to-event analysis was used, and the survival distribution was compared using the log-rank test. Complete case analysis was used to handle missing data.

#### 2.6.3. Analysis of Longitudinal Echocardiographic Data

Linear mixed-effects models were employed to analyze the longitudinal changes in echocardiographic parameters, specifically EF, EDD, and ESD. The models included fixed effects for the type of valve prosthesis, time (as a continuous variable), and the interaction between valve type and time. Random effects were included for both the intercept and the slope of time, allowing each patient to have their own baseline value and trajectory of change over time. An unstructured covariance matrix was used for the random effects to provide maximum flexibility in modeling patient-to-patient variability. The results of the mixed-effects models are presented as coefficients (ß) and 95% confidence intervals.

#### 2.6.4. Sensitivity Analysis

As a sensitivity analysis, we performed inverse probability weighting with regression adjustment (IPWRA) to address potential baseline imbalances between valve groups. This method combines propensity score weighting with outcome regression modeling to estimate potential outcome means for each valve type. We used a parsimonious covariate set including age, diabetes mellitus, hypertension, creatinine clearance, baseline left ventricular end-diastolic dimension, valve size, CPB time, and cross-clamp time to optimize covariate balance and weight stability. The treatment model was specified as a multinomial logistic regression predicting valve group membership, and inverse probability weights were calculated as the reciprocal of the predicted probability of receiving the observed treatment. The outcome model was specified as a linear probability model estimating the risk of the composite endpoint (event occurrence) conditional on the same covariates. Because IPWRA does not account for time-to-event or competing risks, this analysis estimates the overall risk of experiencing the composite endpoint during the follow-up period. Potential outcome means (representing estimated event probabilities for each valve type) and pairwise risk differences were estimated with robust standard errors. Covariate balance was assessed using standardized differences, with values < 0.1 indicating excellent balance and <0.25 indicating acceptable balance. Weight diagnostics were performed to identify extreme weights and assess overlap between treatment groups. Multiple comparisons were adjusted using the Bonferroni correction. All IPWRA analyses were performed using the teffects ipwra command.

## 3. Results

### 3.1. Baseline and Operative Characteristics

The preoperative characteristics of the patient groups are summarized in Table 1. Significant differences were observed among the groups in terms of diabetes prevalence and preoperative creatinine clearance. The Bicarbon group had a slightly older patient population, although this difference did not reach statistical significance, whereas the ATS group had a lower prevalence of diabetes. Creatinine clearance was also noted to differ significantly across the four valve types. The LVEDD was also significantly different between the groups preoperatively. The other preoperative variables were comparable between groups.

Operative data are presented in Table 2. There were statistically significant differences in CPB time, aortic cross-clamp time, and the size of the implanted valve. The ATS group had the longest CPB and aortic cross-clamp times. Valve sizes were also significantly different across the groups, with the On-X group receiving larger valves on average. It is worth noting that while the On-X mitral prosthesis is available in a range of sizes, the Conform-X sewing ring, which was used in some of our patients, is available in a single size (25/33 mm). This may have contributed to the observed differences in average valve size.

### 3.2. Postoperative Outcomes

Early postoperative outcomes are detailed in Table 3. The incidence of re-exploration for bleeding, the amount of red blood cell (RBC) transfusion, and the length of ICU stay were all significantly different among the four groups. The Bicarbon group had the highest rate of re-explorations. The ATS group required more RBC transfusions and had a longer ICU stay. Postoperative pulmonary artery systolic pressure (PASP) also showed significant differences between the groups, with the lowest values recorded in the On-X group. The mean mitral valve gradient did not differ between groups. There were no statistically significant differences in mortality, new-onset atrial fibrillation, stroke, dialysis, LVEF, and hospital stay between groups.

### 3.3. Long-Term Outcomes and Echocardiographic Follow-Up

**Figure 1:**
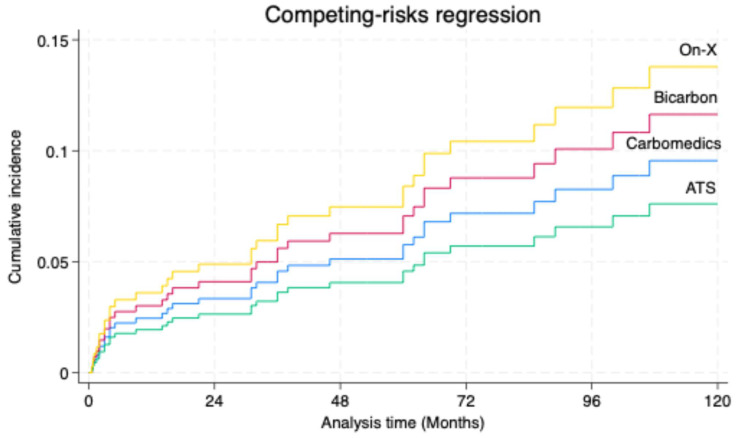
Cumulative incidence of the composite endpoint in the four mechanical valve groups.

The median follow-up time was 62 months (29–109). The incidence rates of the composite endpoint varied across the four study groups. Over a total of 2306.9 person-years of follow-up, 37 events were observed. The highest rate occurred in the Bicarbon group, with 19 events over 934.3 person-years, corresponding to an incidence of 2.03 events per 100 person-years (95% CI: 1.30–3.19). Carbomedics had an incidence of 1.32 events per 100 person-years (95% CI: 0.73–2.39) based on 11 events over 830.9 person-years. ATS and On-X had fewer events and wider confidence intervals: ATS experienced 4 events over 379.4 person-years (rate: 1.05 per 100 person-years; 95% CI: 0.40–2.81), and On-X had 3 events over 162.3 person-years (rate: 1.85 per 100 person-years; 95% CI: 0.60–5.73). The overall incidence rate across all participants was 1.60 events per 100 person-years (95% CI: 1.16–2.21). However, competing risk regression of the composite endpoint, with mortality as a competing event, showed that the cumulative incidence at 10 years was 14% for the On-X valve, 12% for the Bicarbon valve, 9.5% for the Carbomedics valve, and 7% for the ATS valve (Figure 1). Log-rank test indicated no difference in valve thrombosis (*p* = 0.189), stroke (*p* = 0.318), major bleeding (*p* = 0.899), and mitral valve reintervention (*p* = 0.365) between groups. Mitral valve reoperations occurred in 8 patients, 2 in Carbomedics, 5 in Bicarbon, 1 in On-X, and none in the ATS groups. Redo was performed for valve thrombosis in 4 patients and infective endocarditis in 2 patients. The data showed no statistical difference in time to recurrence of NYHA class III or IV between groups (log-rank *p* = 0.272). Freedom from NYHA III/V at 5 years was 95% for Carbomedics, 86% for Bicarbon, 97% for ATS, and 93% for On-X. Heart failure rehospitalization was not statistically different across valve types (log-rank *p* = 0.821). Freedom from HF readmission at 5 years was 96% for Carbomedics, 99% for Bicarbon, 95% for ATS and 100% for On-X.

Time to therapeutic range (TTR) was significantly different between the groups (*p* = 0.002), with the ATS group showing the highest median TTR [58.6% (34.4–77.6)]. TTR was 32.5% (15–54.5) in the Carbomedics group, 44.9% (26.6–70.9) in the Bicarbon group, and 47.6% (42.3–70) in the On-X group.

**Figure 2:**
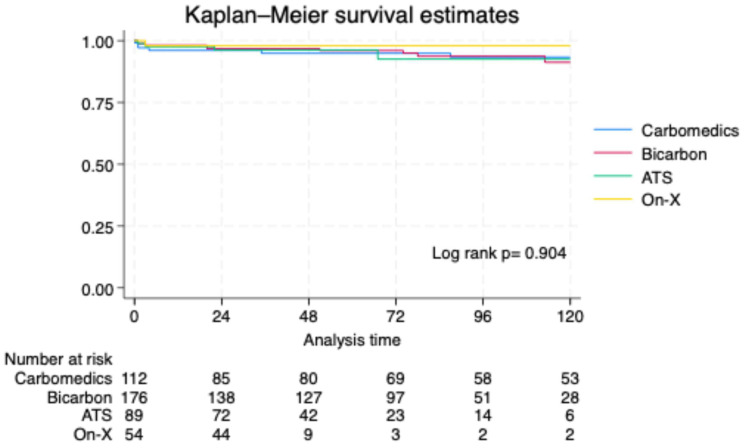
Kaplan–Meier survival curve comparing the four mechanical mitral valves.

A competing risk regression analysis was performed to assess predictors of the composite endpoint (Table 4). After adjusting for confounders, coronary artery disease (CAD), peripheral artery disease (PAD), and smaller valve size were identified as significant predictors of the composite endpoint. The type of valve prosthesis was not significantly associated with the composite endpoint. No statistical difference in survival was reported between groups (log-rank *p* = 0.904) (Figure 2).

Longitudinal analysis of echocardiographic parameters was conducted using mixed-effects models. The trajectory of the LVEF over time did not show significant differences between the valve groups (Table 5). However, the analysis of LVEDD revealed that the Carbomedics group (Group 1), which had the largest baseline LVEDD, showed the most significant improvement in remodeling over time (Table 5). The trajectories of LVESD did not show significant differences between the groups (Table 5).

### 3.4. Sensitivity Analysis: Inverse Probability Weighting

As a sensitivity analysis, we performed IPWRA using a parsimonious covariate set to optimize covariate balance. The analysis included 390 patients and adjusted for 8 key confounders. The potential composite outcome means were 24.2% (95% CI: 11.0–37.5%) for Carbomedics, 14.9% (95% CI: 7.6–22.2%) for Bicarbon, 12.7% (95% CI: 1.1–24.2%) for ATS, and 11.1% (95% CI: -4.8–26.9%) for On-X. However, pairwise comparisons with Bonferroni correction revealed no statistically significant differences among valve types (all adjusted *p*-values > 0.99). The risk differences ranged from −9.3% to −13.2% when comparing other valves to Carbomedics, but all confidence intervals included zero (Table 6). Balance diagnostics showed acceptable covariate balance, with most standardized differences < 0.2 after weighting. Inverse probability weights ranged from 1.02 to 38.48 (mean: 4.08), indicating moderate overlap between treatment groups. These findings are consistent with our primary competing-risk regression analysis.

## 4. Discussion

This study provides a comprehensive, long-term comparison of four contemporary bileaflet mechanical mitral valve prostheses. The principal finding of our analysis is that after a median follow-up of 62 months, there were no statistically significant differences in the composite endpoint of major adverse events or overall survival among the Carbomedics, Bicarbon, ATS, and On-X valves. This suggests that, in terms of thromboembolic, bleeding complications, and valve reoperation, the four prostheses have a comparable safety profile. Our findings are consistent with the general consensus in the literature that modern bileaflet mechanical valves have similar and excellent long-term outcomes [9,14,15,16].

Bileaflet valves are now the most commonly used; they offer good effective orifice area, relatively low transvalvular gradients, and excellent durability [17]. In a randomized controlled comparison of On-X vs St Jude mechanical prostheses, including the mitral position, haemodynamic performance and valve-related event rates were essentially equivalent; the study did not demonstrate a lower thromboembolic rate with On-X under standard INR targets [18]. Echocardiographic studies in the mitral and aortic positions suggest On-X valves may have slightly larger effective orifice area and marginally lower gradients in some sizes, but these differences have not translated into clearly superior clinical outcomes to date [19]. Studies comparing the long-term outcomes among various bileaflet mitral valves are scarce. The absence of a significant difference in the composite endpoint across valve types underscores that patient-related factors and the quality of anticoagulation management are likely more important determinants of long-term outcomes than the specific mechanical prosthesis design [20]. In our competing risk regression analysis, we identified coronary artery disease, peripheral artery disease, and smaller valve size as independent predictors of adverse events. The association between established atherosclerotic disease and a higher risk of thromboembolic events is well-documented and highlights the importance of aggressive secondary prevention in this patient population [21]. The finding that smaller valve size is a predictor of adverse events is also consistent with previous studies and is likely related to the higher transvalvular gradients and increased turbulence associated with smaller prostheses, which can promote thrombus formation [22].

An important consideration in interpreting these findings is the potential for selection bias. The choice of valve prosthesis in our study was not randomized but rather determined by surgeon preference, institutional contracts, availability, and patient-specific anatomical considerations. The observed differences in operative characteristics among the groups, specifically, the larger valve sizes in the On-X group and the longer CPB and cross-clamp times in the ATS group, suggest that these valves may have been preferentially selected for patients with different anatomical or pathological profiles. For instance, surgeons may have chosen certain prostheses for patients with larger or smaller annuli, more extensive calcification, or more complex surgical scenarios. While we attempted to adjust for measured confounders through multivariable analysis, unmeasured differences in patient selection could still influence the observed outcomes. This inherent limitation of observational studies underscores the need for caution when interpreting comparisons between valve types.

An interesting finding of our study is a significant difference in anticoagulation control quality, as measured by TTR, among the four valve groups. The ATS group had the highest median TTR, which may have contributed to the numerically lower, albeit not statistically significant, incidence of the composite endpoint in this group. The reasons for this difference in TTR are not immediately clear from our data, but could be related to subtle differences in patient characteristics or anticoagulation management protocols that were not captured in our retrospective analysis [23]. This finding warrants further investigation, as optimizing TTR is a critical component of care for patients with mechanical valves [24,25]. TTR does not reflect anticoagulation control throughout the follow-up period, and INR values may not be related to the time of occurrence of the composite endpoint. Therefore, it is not surprising that TTR was not a significant predictor for the outcomes in our series. To overcome the drawback of warfarin, several studies have been conducted to replace or reduce the dose of warfarin in managing anticoagulation for mechanical mitral valves, but the results were inconclusive [26,27,28,29].

Another important finding of our study is the differential impact of the valve types on left ventricular remodeling. The Carbomedics group, which had the largest baseline left ventricular end-diastolic dimension, demonstrated the most significant improvement in remodeling over time. This suggests that the hemodynamic performance of the Carbomedics valve may be particularly favorable in patients with significant left ventricular dilation at the time of surgery. The mechanisms underlying this observation are likely multifactorial and may involve differences in the effective orifice area, transvalvular gradients, and flow dynamics of the different prostheses. Furthermore, the changes could be attributed to patients’ characteristics not captured in our study [30,31]. The greater LV dimensional reduction observed in the Carbomedics group should be interpreted cautiously. This group had significantly larger baseline LVEDD, and the magnitude of reduction was proportional to baseline severity. This pattern is consistent with regression to the mean, whereby extreme baseline values tend to normalize upon repeat measurement, rather than a prosthesis-specific effect. All valve types appear to provide adequate hemodynamic relief for favorable reverse remodeling. Furthermore, the clinical significance of this finding is not yet clear; it raises the intriguing possibility that, in the future, the choice of mechanical prosthesis could be tailored to the specific anatomical and physiological characteristics of the patient.

The IPWRA sensitivity analysis corroborated our primary findings, showing no statistically significant differences in outcomes among the four valve types. While the point estimates suggested a trend toward lower risk with Bicarbon, ATS, and On-X compared to Carbomedics (risk differences: −9.3% to −13.2%), the wide confidence intervals (all including zero) and non-significant p-values (all > 0.19) indicate that these differences are not statistically reliable. The consistency between the IPWRA and competing-risk regression analyses, despite different methodological approaches, strengthens confidence in the conclusion that modern mechanical mitral valves perform comparably in clinical practice.

### Limitations

Our study has several limitations that should be acknowledged. First, as a retrospective, single-center study, it is subject to the inherent biases of this study design. Although we used multivariable regression and competing risk analysis to adjust for confounders, we cannot exclude the possibility of unmeasured confounding variables. The differences in surgical details, specifically regarding subvalvular apparatus and posterior leaflet preservation between groups, were not reported, and these details could have affected the outcomes. Second, selection bias is an important limitation, as valve choice was not randomized but based on surgeon preference, institutional factors, and anatomical considerations. The observed differences in valve sizes and operative times among groups suggest that certain prostheses may have been preferentially used in specific clinical scenarios, potentially confounding the comparison despite multivariable adjustment. Third, a post hoc power analysis was not performed. Given the retrospective nature of the study and the relatively low number of adverse events observed, the study may be underpowered to detect small but clinically meaningful differences between the valve types. This is particularly true for the On-X group, which had the smallest sample size. Therefore, the non-significant findings should be interpreted with caution, as the possibility of a type II error cannot be excluded. Fourth, the follow-up was not 100% complete, and some events may have been missed. Fifth, longitudinal prosthetic valve gradient data were not consistently available, limiting our ability to link remodeling patterns to hemodynamic performance. However, postoperative gradients were comparable across valve types, and remodeling magnitude was proportional to baseline severity rather than valve type, suggesting differential hemodynamics are unlikely to explain the observed patterns. Finally, the quality of anticoagulation control was assessed by TTR, which is a widely used but imperfect measure of anticoagulation quality. Furthermore, it was not available for all patients.

## 5. Conclusions

Our study demonstrates that the four contemporary bileaflet mechanical mitral valve prostheses investigated have a comparable and excellent long-term safety profile. The choice of prosthesis does not appear to significantly influence the risk of major adverse valve-related events or overall survival. Patient-related factors, such as the presence of atherosclerotic disease and smaller valve size, are the primary determinants of adverse outcomes. The observed differences in left ventricular remodeling among the valve types are intriguing and warrant further investigation in larger, prospective studies. Our findings support the current practice of selecting a mechanical mitral valve prosthesis based on surgeon preference and availability, rather than on the expectation of a superior long-term outcome with a specific valve type.

## Figures and Tables

**Table 1:** Comparison of the preoperative characteristics between four mechanical mitral valves.

Variable	Carbomedics (n = 112)	Bicarbon (n = 176)	ATS (n = 89)	On-X (n = 54)	*p*-Value
Age (years), median [IQR]	48.0 [41.0–54.5]	51.0 [43.0–56.0]	46.0 [40.0–54.0]	50.0 [45.0–56.0]	0.060
Gender (Male), n (%)	52 (46.4%)	71 (40.3%)	38 (42.7%)	24 (44.4%)	0.779
Body Mass Index (BMI, kg/m^2^), median [IQR]	27.3 [23.7–32.3]	28.6 [24.2–32.9]	28.7 [25.1–32.3]	28.8 [24.7–32.6]	0.643
Smoking, n (%)	12 (10.7%)	21 (11.9%)	9 (10.1%)	6 (11.1%)	0.973
EuroSCORE II (%), median [IQR]	4.08 [2.33–6.30]	3.03 [1.71–5.34]	2.99 [1.84–6.12]	2.43 [2.00–4.72]	0.064
Diabetes Mellitus, n (%)	37 (33.0%)	67 (38.1%)	18 (20.2%)	13 (24.1%)	0.016
Hypertension, n (%)	51 (45.5%)	65 (36.9%)	27 (30.3%)	16 (29.6%)	0.091
Atrial Fibrillation (Afib), n (%)	48 (42.9%)	78 (44.3%)	49 (55.1%)	26 (48.1%)	0.307
History of Myocardial Infarction, n (%)	7 (6.3%)	8 (4.5%)	2 (2.2%)	1 (1.9%)	0.500
History of Stroke, n (%)	9 (8.0%)	16 (9.1%)	7 (7.9%)	9 (16.7%)	0.283
Coronary Artery Disease, n (%)	7 (6.3%)	23 (13.1%)	6 (6.7%)	6 (11.1%)	0.188
Heart Failure, n (%)	11 (10.3%)	14 (8.1%)	5 (5.7%)	4 (7.5%)	0.706
Chronic kidney disease, n (%)	5 (4.5%)	10 (5.7%)	1 (1.1%)	4 (7.4%)	0.234
Anemia, n (%)	35 (31.2%)	58 (33.0%)	35 (39.3%)	17 (31.5%)	0.631
Peripheral Artery Disease, n (%)	2 (1.8%)	3 (1.7%)	0 (0.0%)	1 (1.9%)	0.620
Thrombocytopenia, n (%)	8 (7.1%)	15 (8.5%)	7 (7.9%)	3 (5.6%)	0.950
Creatinine Clearance (mL/min), median [IQR]	115.2 [92.1–153.5]	104.1 [81.5–129.9]	102.6 [83.9–123.3]	100.8 [78.3–120.8]	0.007
Preoperative Bilirubin (μmol/L), median [IQR]	9 [6–14]	10 [6–15]	10 [7–15]	7.5 [5–14]	0.543
Ejection fraction (%), median [IQR]	55 [45–55]	55 [50–55]	55 [50–55]	55 [50–55]	0.884
End-diastolic diameter (mm), median [IQR]	53 [47–59]	51 [46–57]	49 [44–56]	50 [46–54]	0.026
End-systolic diameter (mm), median [IQR]	34.5 [30–42]	34 [29–40]	34.5 [30–39]	34 [29–40]	0.526
Mean mitral pressure gradient (mmHg), median [IQR]	7.6 [5.3–12]	7.9 [5.9–12.3]	8.9 [6–11.8]	8.8 [6.3–11.6]	0.575
Mitral stenosis, n (%)	64 (58.2%)	100 (57.1%)	56 (63.6%)	33 (66.0%)	0.578
Pulmonary artery systolic pressure (mmHg), median [IQR]	50 [40–62.5]	50 [40–60]	45 [40–60]	45 [35–55]	0.063
Right ventricle dysfunction, n (%)	15 (13.5%)	29 (16.5%)	15 (16.9%)	5 (10.0%)	0.634

Data are presented as n (%) for categorical variables and median [Interquartile Range] or mean (Standard Deviation) for continuous variables. *p*-values are from chi-squared/Fisher’s Exact Test for categorical variables and Kruskal–Wallis or ANOVA for continuous variables.

**Table 2:** Comparison of the operative characteristics between four mechanical mitral valves.

Variable	Carbomedics (n = 112)	Bicarbon (n = 176)	ATS (n = 89)	On-X (n = 54)	*p*-Value
Redo Surgery, n (%)	46 (41.4%)	61 (34.7%)	36 (40.5%)	15 (30.0%)	0.415
Urgent/ emergency, n (%)	1 (0.9%)	3 (1.7%)	1 (1.1%)	2 (4.0%)	0.537
Concomitant, n (%)	78 (69.6%)	130 (73.9%)	56 (62.9%)	31 (57.4%)	0.080
Cardiopulmonary Bypass Time (min), median [IQR]	125 [102–157]	123 [93–157.5]	146 [112–187]	134 [114–168]	0.003
Aortic Cross-Clamp Time (min), median [IQR]	94 [67–112]	94 [70–124]	106 [85–145]	98.5 [77–126]	0.003
Valve size (mm), median [IQR]	27 [25–29]	25 [25–27]	27 [25–27]	27 [27–33]	<0.001

Data are presented as n (%) or median [Interquartile Range]. *p*-values are from chi-squared/Fisher’s Exact Test or Kruskal–Wallis Test.

**Table 3:** Comparison of the post-operative outcomes between four mechanical mitral valves.

Variable	Carbomedics (n = 112)	Bicarbon (n = 176)	ATS (n = 89)	On-X (n = 54)	*p*-Value
Early Mortality, n (%)	4 (3.6%)	4 (2.3%)	3 (3.4%)	0 (0.0%)	0.599
Valve Thrombosis, n (%)	0 (0.0%)	1 (0.6%)	0 (0.0%)	0 (0.0%)	>0.99
Atrial fibrillation, n (%)	4 (6.7%)	15 (8.5%)	8 (9.1%)	4 (8.3%)	0.961
Stroke, n (%)	2 (1.8%)	2 (1.1%)	0 (0.0%)	0 (0.0%)	0.687
Re-exploration, n (%)	4 (3.7%)	17 (9.8%)	0 (0.0%)	1 (1.9%)	0.002
Cardiac Tamponade, n (%)	0 (0.0%)	2 (1.1%)	0 (0.0%)	0 (0.0%)	0.620
RBCS transfusion (units), median [IQR]	2 [2–4]	2 [1–5]	5 [2–6]	3 [1–4]	0.034
FFP transfusion (units), median [IQR]	5 [2.5–9]	6 [3.5–6.5]	5 [4–6]	4 [4–6]	0.898
Platelets transfusion (units), median [IQR]	4 [1.5–6]	5 [2–6]	1 [1–4]	2 [1–4]	0.061
Dialysis/CRRT, n (%)	2 (3.4%)	2 (1.2%)	2 (2.3%)	0	0.503
ICU Stay (days), median [IQR]	1.5 [1.0–3.0]	2.0 [1.0–4.0]	3.0 [2.0–5.0]	3.0 [2.0–5.0]	<0.001
Hospital Stay (days), median [IQR]	9.0 [7.0–16.0]	12.0 [8.0–18.5]	12.0 [9.0–18.0]	12.0 [8.0–16.0]	0.098
Ejection fraction (%), median [IQR]	55 [40–55]	55 [45–55]	55 [50–55]	55 [50–55]	0.253
EDD (mm), median [IQR]	50 [45–55]	48 [44–52]	46 [41–53]	47 [42–51]	0.073
ESD (mm), median [IQR]	35 [29–41]	33 [29–39]	32 [27–39]	33 [30–38]	0.390
Mean mitral valve pressure gradient (mmHg), median [IQR]	5.3 [4–6.8]	4.8 [3.8–6.6]	5 [4.1–6.2]	5 [4.2–6]	0.489
PASP (mmHg), median [IQR]	35 [30–45]	40 [35–45]	40 [30–45]	30 [25–40]	0.014

CRRT: continuous renal replacement therapy; EDD: end-diastolic diameter; ESD: end-systolic diameter; FFP: fresh frozen plasma; ICU: intensive care unit, PASP: pulmonary artery systolic pressure, RBCs: red blood cells. Data are presented as n (%) or median [Interquartile Range]. *p*-values are from chi-squared/Fisher’s Exact Test or Kruskal–Wallis Test.

**Table 4:** Competing risk regression for composite endpoint in the presence of death as a competing risk.

Variable	SHR (95% CI)	*p*-Value
Valve type (ref: Carbomedics)		
Bicarbon	1.23 [0.60–2.55]	0.572
ATS	0.79 [0.25–2.50]	0.686
On-X	1.48 [0.39–5.54]	0.563
Coronary artery disease	2.70 [1.15–6.35]	0.023
Peripheral Artery Disease	6.29 [1.66–23.79]	0.007
Valve Size	0.87 [0.77–0.99]	0.037

**Table 5:** Mixed-effects model for ejection fraction, end-diastolic dimension, and end-systolic dimension trajectories.

	ß (95% CI)	*p*-Value
Ejection fraction Carbomedic (Ref) Bicarbon ATS On-X Time Bicarbon#Time ATS#Time On-X#Time	0.623 (−1.488 to 2.734) 1.264 (−1.217 to 3.745) 2.684 (−0.291 to 5.66) −0.004 (−0.036 to 0.028) −0.004 0.021 −0.065	0.563 0.318 0.077 0.816 0.816 0.351 0.094
End-diastolic diameter Carbomedic (Ref) Bicarbon ATS On-X Time Bicarbon#Time ATS#Time On-X#Time	−2.671 (−4.48 to −0.862) −3.507 (−5.636 to −1.377) −2.819 (−5.357 to −0.28) −0.042 (−0.062 to −0.021) 0.031 (0.004 to 0.057) 0.033 (−0.004 to 0.071) 0.018 (−0.047 to 0.083)	0.004 0.001 0.03 <0.001 0.024 0.08 0.581
End-systolic diameter Carbomedic (Ref) Bicarbon ATS On-X Time Bicarbon#Time ATS#Time On-X#Time	−1.684 (−3.657 to 0.28) −1.894 (−4.22 to 0.432) −1.654 (−4.434 to 1.127) −0.02 (−0.042 to 0.001) 0.019 (-0.01 to 0.047) −0.012 (−0.052 to 0.029) 0.029 (−0.045 to 0.103)	0.094 0.11 0.244 0.067 0.206 0.565 0.445

**Table 6:** Pairwise comparison of the risk difference in various valve types for the composite endpoint.

Comparison	Risk Difference (95% Confidence Interval)	*p*-Value	Bonferroni *p*
Bicarbon vs. Carbomedics	−9.3 (−24.4–5.8)	0.225	>0.99
ATS vs. Carbomedics	−11.6 (−29.2–6.0)	0.198	>0.99
On-X vs. Carbomedics	−13.2 (−33.9–7.6)	0.214	>0.99
ATS vs. Bicarbon	−2.2 (−20.4–16.0)	>0.99	>0.99
On-X vs. Bicarbon	−3.8 (−27.3–19.7)	>0.99	>0.99
On-X vs. ATS	−1.6 (−28.1–24.9)	>0.99	>0.99

## Data Availability

Data sharing requires a data sharing agreement in accordance with institutional regulations.

## Abbreviations

The following abbreviations are used in this manuscript:

INR	International Normalized Ratio
CAD	Coronary Artery Disease
PAD	Peripheral Artery Disease
ICU	Intensive Care Unit
RBC	Red Blood Cell
FFP	Fresh Frozen Plasma
CRRT	Continuous Renal Replacement Therapy
MVR	Mitral Valve Replacement
LVEF	Left Ventricular Ejection Fraction
LVEDD	Left Ventricular End-Diastolic Dimension
LVESD	Left Ventricular End-Systolic Dimension
EF	Ejection Fraction
EDD	End-Diastolic Diameter
ESD	End-Systolic Diameter
PASP	Pulmonary Artery Systolic Pressure
SHR	Sub-distribution Hazard Ratio
CI	Confidence Interval
IQR	Interquartile Range
SD	Standard Deviation
TTR	Time in Therapeutic Range
ANOVA	Analysis of Variance
STROBE	Strengthening the Reporting of Observational Studies in Epidemiology
EuroSCORE	European System for Cardiac Operative Risk Evaluation
BARC	Bleeding Academic Research Consortium
